# Vision Impairment Among the Jirel Population of Nepal

**DOI:** 10.1001/jamanetworkopen.2025.27812

**Published:** 2025-08-25

**Authors:** Karthik Reddy, Bhaskar Jha, Pradeep Banjara, Manish Poudel, Mohan K. Shrestha, Anup Joshi, Joshua R. Ehrlich, Suman Thapa

**Affiliations:** 1 University of Michigan Medical School, Ann Arbor; 2 Department of Ophthalmology and Visual Sciences, University of Michigan, Ann Arbor; 3 Tilganga Institute of Ophthalmology, Kathmandu, Nepal; 4 Survey Research Center, Institute for Social Research, University of Michigan, Ann Arbor

## Abstract

This cross-sectional study reports the prevalence of vision impairment amount the Jirel people, an understudied Indigenous group in Nepal.

## Introduction

Indigenous populations face disproportionately high rates of preventable eye diseases.^1^ Nepal’s prevalence of vision impairment (VI) is 20.7%, with inequities in disease burden and treatment access compared with other Asian populations.^2,3^ Because Indigenous groups like the Jirel people remain understudied, it is vital to identify which subpopulations contribute to Nepal’s high overall rate of VI.^1^ The Jirel people are an indigenous, rural minority uniquely vulnerable given their intersection of religious and ethnic minority status and residence in resource-limited settings. This study estimates the prevalence of VI and associated factors among the Indigenous Jirel people.

## Methods

This population-based, cross-sectional study was conducted in the Jiri municipality of the Dolakha district (eMethods in Supplement 1).^4^ VI was defined as mild (<6/12 to 6/18), moderate (<6/18 to 6/60), severe (<6/60 to 3/60), or blindness (<3/60). From March 2015 to December 2018, 2042 Jirel patients were recruited from 7 Jirel villages utilizing a convenience sampling protocol. Informed consent was obtained from participants by investigators on the day of their appointment. Demographic information was collected, and a comprehensive ophthalmic evaluation was performed. Descriptive statistics, prevalence, and associated factors were calculated across all groups with 95% CIs. Statistical significance (2-tailed) was set at *P* < .05, and data were analyzed using SPSS (IBM SPSS Statistics for Windows, version 20). The Nepal Health Research Council and the University of Texas Health Science Center Institutional Review Board approved this study. The study followed STROBE guidelines.

## Results

A total of 2042 patients were enrolled in the study, including 1105 females (54.1%) and 900 illiterate individuals (44.1%). Ages ranged from 18 to 88 years, with a mean (SD) age of 42.3 (16.7) years. The prevalence of any VI (<6/12) on presentation for the total population was 13.2% (95% CI, 11.7%-14.6%) and 4.1% (95% CI, 3.3%-5%) after best correction. On presentation, 86.8% had no VI, 4.6% had mild VI, 8.0% had moderate VI, 0.2% had severe VI, and 0.3% were blind. Refractive error was the most common cause of VI (68.9% of VI on presentation). The prevalence of VI stratified by age is presented in the Figure.

**Figure.**
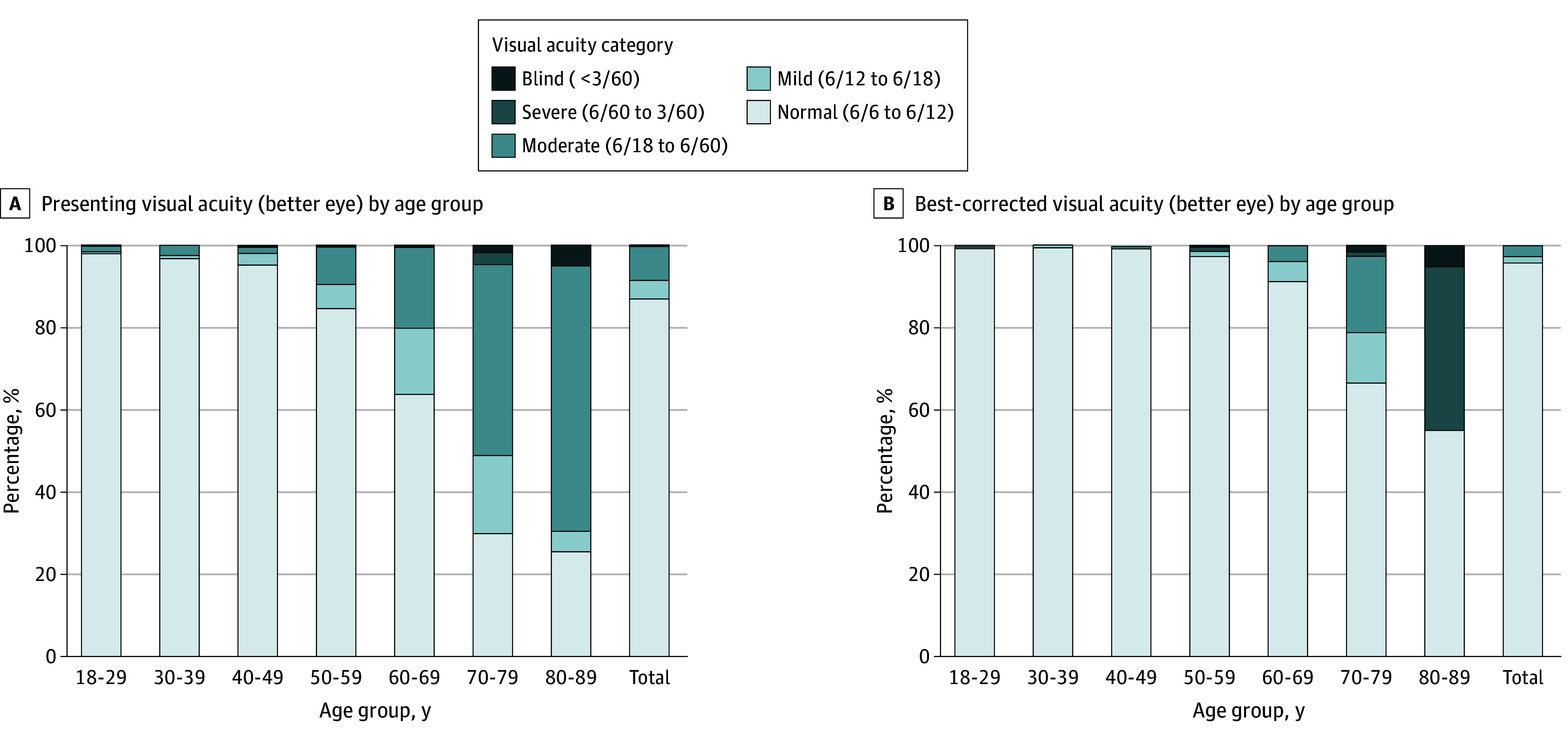
Prevalence and Type of Vision Impairment Among the Jirel Population on Presentation and After Best Correction by Age Group Visual impairment was categorized based on *International Classification of Diseases* criteria.

For those aged 40 years and older (n = 1044), the prevalence of any VI (<6/12) on presentation was 23.2% (95% CI, 20.6%-25.7%) and 7.5% (95% CI, 5.9%-9.1%) after best correction. On presentation for this group, 76.8% had normal vision, 8.3% had mild VI, 14.0% had moderate VI, 0.3% had severe VI, and 0.6% were blind. On bivariate analysis, individuals (≥40 years) with VI were 44.9% male and 55.1% female, similar to those without VI (48.8% male and 51.2% female; *P* = .74). Compared with those with normal vision, individuals with VI (≥40 years) were significantly more likely to be illiterate (93.9% vs 70.2%; *P* < .001). On multivariate analysis, every additional year older than 40 years was significantly associated with a 0.007 logMAR increase in VI (SE = 0.003; *P* = .02). All factors associated with VI in the multivariate analysis are presented in the Table.

**Table.** Associated Factors of Vision Impairment Among the Jirel Population Aged 40 and Above: Multivariate Analyses

Variable	Change in vision (logMAR) (SE)	*P* value	OR (95% CI)
Age, y	0.007 (0.003)	.02	1.01 (1.00 to 1.01)
Sex (reference group, male)	0.082 (0.322)	.80	1.09 (0.58 to 2.04)
BMI	−0.157 (0.048)	.001	0.86 (0.78 to 0.94)
Smoking (reference group, no smoking)		NA	NA
Current smoking	0.511 (0.389)	.19	1.67 (0.78 to 3.58)
Former smoking	−0.081 (0.423)	.85	0.92 (0.40 to 2.11)
Alcohol (reference group, no alcohol use)	NA	NA	NA
Current alcohol use	−0.008 (0.488)	.98	0.99 (0.38 to 2.58)
Former alcohol use	0.645 (0.543)	.24	1.91 (0.66 to 5.53)
Systemic illness (reference group, no systemic illness)	0.085 (0.317)	.79	1.09 (0.59 to 2.03)
Nonagricultural occupation (reference group, agricultural occupation or farmer)	1.335 (0.322)	<.001	3.80 (2.02 to 7.14)

Abbreviation: logMAR, logarithm of the minimum angle of resolution; OR, odds ratio.

## Discussion

In this study, 13.2% of all Jirel people presented with VI; among individuals aged 40 years and older, the prevalence was 23.2%. Uncorrected refractive error was the most common cause of VI among the total population. Those who were older and illiterate were more likely to have VI. These results suggest that the Jirel population continues to experience higher rates of VI (<6/12 in the better eye) compared with national estimates (20.7% with VI among those aged ≥50 years) and markedly higher than urban regions.^2,5,6^ This disparity exists despite outreach since 2010 and the establishment of a low-cost community eye center in 2015.

The burden of VI among the Indigenous Jirel population may further be underestimated. This is because of possible insufficient outreach in inaccessible enclaves and patient unwillingness to undergo screening, a common phenomenon among communities with a stigma attached to blindness. Because we included participants aged 40 to 49 years with lower rates of VI than those aged 50 years and older, our overall estimates may appear lower than in national estimates restricted to individuals aged 50 years and older.^2^

This highlights the need for further interventions to reduce the prevalence of VI among the Jirel people, such as easily treated simple refractive error, and to characterize VI among other Indigenous populations in South Asia. Limitations included participants’ literacy or cultural beliefs limiting examination and the inaccessibility of some small enclaves of the Jirel people. However, this study illustrates the continued importance of quantifying VI at regional levels and among marginalized communities.

## Notes

Supplement 1.
eMethods.

eReferences.

Supplement 2.
Data Sharing Statement
