# Supplementary material for: Vision Impairment Among the Jirel Population of Nepal
Source: JAMA Netw Open. 2025 Aug 25;8(8):e2527812. doi: 10.1001/jamanetworkopen.2025.27812 (PMC12379085; doi:10.1001/jamanetworkopen.2025.27812)
Supplement: Supplement 1. — eMethods. eReferences. [file jamanetwopen-e2527812-s001.pdf]

Supplemental Online Content

Reddy K, Jha B, Banjara P, et al. Vision impairment among the Jirel population of Nepal. *JAMA Netw Open.* 2025;8(8):e2527812.
doi:10.1001/jamanetworkopen.2025.27812

Supplement 1. eMethods

eReferences.

This supplemental material has been provided by the authors to give readers additional information about their work.

© 2025 Reddy K et al. *JAMA Network Open*

Field Work

The study participants belong to a single extended pedigree, which has been utilized for many prior genetic epidemiological studies.[1] The Jiri Eye Study (JES) was initially conceived as the first study to assess the heritability of ocular traits in an Indigenous Nepali population, for which a sample size of 2,000 was deemed appropriate by power analysis.[2] Criteria for inclusion included individuals belonging to the Jirel ethnic community, who were at least 18 years of age, and in good health.

The Nepal Health Research Council and Texas Biomed (the University of Texas Health Science Center at San Antonio Institutional Review Board [IRB]) approved this study. The study included individuals 18 years or older. Subject recruitment and subsequent ocular examination were performed during biannual visits to the field site (250 subjects per visit, 500 subjects per year). Several local recruiters contacted village elders and community leaders in Jirel communities surrounding Jiri to notify their community members of the upcoming study. Additionally, a list of Jirel people who were participants in prior genetic studies[1,3] was used to contact individuals from the seven surrounding villages. Three weeks before each field study, a meeting was held in the village of Hatdada within the Jiri municipality. All Jirel people from the village(s) targeted for inclusion were invited to participate. One week later, local recruiters contacted sampled individuals, providing an appointment within the two-week fieldwork period of the JES.

Patient examinations were conducted at a local ophthalmology clinic established for the JES. Investigators obtained participants' consent on the day of their appointment and provided

additional information as needed. Consent was documented by signature or fingerprint as approved by the Nepal Health Research Council and Texas Biomed (the University of Health Science Center at San Antonio IRB).

Given Nepal's high illiteracy rate, a witness was also made to sign the consent form for illiterate individuals who documented their consent with a fingerprint.[4] Literacy was determined by self-reported level of educational attainment for the purpose of analysis,[5] with those receiving no formal education categorized as not literate.

Data Collection

Detailed interviews were conducted. The interviews included medical and ocular histories, with subsequent ophthalmic evaluation. The general medical history focused on systemic disease(s) such as hypertension, diabetes, or cancer and risk factor questions such as alcohol or tobacco use. The ocular history pertaining to a previously diagnosed eye condition or disease, eyeglass use, eye drop utilization, history of eye trauma, or other known eye problems.

Evaluation included assessing participants' distance and near visual acuity (VA) for both presenting vision and best corrected after refraction. This was measured using a logarithm of the minimum angle of resolution (log MAR) tumbling E charts (Precision Vision, USA) placed at 4 meters. Objective refraction was done using a streak retinoscope (Heine Beta 200, Germany) by trained ophthalmic assistants from the Tilganga Institute of Ophthalmology (TIO), followed by subjective refraction. VI was defined in concordance with the proposed International Statistical Classification of Diseases revision by Dandona et al.[6] The log MAR chart was moved to 1 meter if the subject could not read the top line, and VA was reevaluated. If VA could not be measured, counting fingers at 1 meter, detecting hand movement, or detecting light perception were sequentially assessed. Further, all participants received an evaluation with a slit-lamp

© 2025 Reddy K et al. *JAMA Network Open*

biomicroscope (Haag Streit BQ 900, Germany) by trained ophthalmologists from TIO. A measure of intraocular pressure was also obtained (Goldmann application tonometry). The peripheral anterior chamber was graded using the van Herrick method and gonioscopy using a 4-mirror gonioscope (Zeiss, Germany).[7] The angle was graded according to the Shaffer system.[8]

All subjects subsequently underwent anterior segment photography and OCT (Topcon, Japan) by trained ophthalmic assistants from TIO. Examination assessed corneal thickness, keratometry, iris thickness, and angle opening distance. Automated visual field testing was performed using the SITA Standard 24-2 program (Humphrey Instruments Model 750, USA). Patients without risk for glaucoma underwent subsequent pupillary dilation with fundus photography and posterior OCT to assess the nerve fiber layer and central macular thickness (Topcon, Japan). All other subjects underwent posterior segment examination without the use of dilation. Ocular biometry measurements (NIDEK AL-scan, USA) were obtained to document length and lens thickness. All participants with cataracts were graded by the Lens Opacities Classification System III (LOCS III).[9] Stereoscopic fundus examination was performed using a 90-diopter or 20-diopter lens when necessary.

Data Analysis

Data was imported for statistical analysis in SPSS (IBM SPSS Statistics for Windows, Version 20 (IBM Corp., Armonk, NY, USA). Data was analysed in early 2025. Descriptive statistics were calculated using standard deviation, and prevalence was calculated using condition, level of VI, and demographic factors. The difference in prevalence between types of VI (blind, VI, no impairment) was calculated across age groups using Chi-Square tests.

In assessing risk factors, binary and multivariable logistic regressions were used to calculate VI's associations across individuals 40 and above at 95% confidence intervals (CI). All variables from

© 2025 Reddy K et al. *JAMA Network Open*

the bivariate analysis were evaluated for inclusion in the multiple logistic regression model; however, the “literate” variable was excluded because only three cases fell into the low vision/blind category, producing an excessively high standard error and indicating an insufficient sample size for that group. The pattern of missingness was evaluated by comparing age and sex distributions between complete and incomplete cases, with no significant differences detected, supporting the assumption of missing completely at random. A list-wise deletion strategy was employed. No substantive association was found between variables (maximum correlation of 0.54), which was below concerning levels. Of the 2,042 observations, only 15 (0.7%) were missing vision measurements (Presenting Visual Acuity or Best-corrected Visual Acuity), while all other variables were complete. Statistical significance was set at $p < 0.05$. Individuals with mild or worse VI were grouped into a broader “any VI” category for some analyses.

© 2025 Reddy K et al. *JAMA Network Open*

eReferences

1. Williams-Blangero S, Blangero J. Collection of pedigree data for genetic analysis in isolate populations. *Human Biology*. 2006;78(1):89-101.
2. Johnson MP, Thapa SS, Laston S, et al. Genetic research on ocular health and disease in a population from Nepal. Advances in Vision Research, Volume II: Genetic Eye Research in Asia and the Pacific. Springer; 2018:75–84.
3. Prasai DR. Issues of reservation and affirmative action for minorities in Nepal: An anthropological review. *Himalayan Journal of Sociology and Anthropology*. 2016;7:1-12.
4. Dhakal B. Statistical trends in literacy rates in Nepal. *IOSR Journal of Applied Chemistry*. 2018;11(11):71-77.
5. Park H, Kyei P. Literacy gaps by educational attainment: A cross-national analysis. *Social Forces*. 2011;89(3):879–904.
6. Dandona L, Dandona R. Revision of visual impairment definitions in the International Statistical Classification of Diseases. *BMC medicine*. 2006;4:1-7.
7. Van Herick W, Shaffer RN, Schwartz A. Estimation of width of angle of anterior chamber: incidence and significance of the narrow angle. *American journal of ophthalmology*. 1969;68(4):626-629.
8. Chan RY, Smith JA, Richardson KT. Anterior segment configuration correlated with Shaffer's grading of anterior chamber angle. *Archives of Ophthalmology*. 1981;99(1):104-107.
9. Chylack LT, Wolfe JK, Singer DM, et al. The lens opacities classification system III. *Archives of ophthalmology*. 1993;111(6):831-836.

© 2025 Reddy K et al. *JAMA Network Open*
