# Supplementary material for: Vision Impairment Among the Jirel Population of Nepal
Source: JAMA Netw Open. 2025 Aug 25;8(8):e2527812. doi: 10.1001/jamanetworkopen.2025.27812 (PMC12379085; doi:10.1001/jamanetworkopen.2025.27812)
Supplement: Supplement 2. — Data Sharing Statement [file jamanetwopen-e2527812-s002.pdf]

Data Sharing Statement

Reddy. Vision Impairment Among the Jirel Population of Nepal. *JAMA Netw Open*. Published August 20, 2025. doi:10.1001/jamanetworkopen.2025.27812

Data
Data available: Yes
Data types: Deidentified participant data
How to access data: Please send all requests to Suman Thapa (sumanthapa.ekekpaila@gmail.com).
When available: With publication

Supporting Documents
Document types: Statistical/analytic code, Informed consent form
How to access documents: Please send all requests to Suman Thapa (sumanthapa.ekekpaila@gmail.com).
When available: With publication

Additional Information
Who can access the data: Anyone requesting the data.
Types of analyses: For any purpose.
Mechanisms of data availability: After approval of a proposal.
